# Enhanced Photoluminescence in a Neuromorphic 2D Memitter Based on WS_2_ via Plasmonic Nanoparticle Self-Assembly

**DOI:** 10.1021/acsami.5c03059

**Published:** 2025-06-05

**Authors:** Federico Ferrarese Lupi, Gianluca Milano, Angelo Angelini, Mateo Rosero-Realpe, Irdi Murataj, Bruno Torre, Eleonora Cara, Philipp Hönicke, André Wählisch, Erika Kozma, Diego Antonioli, Michele Laus, Alessia Motta, Christian Martella, Carlo Grazianetti

**Affiliations:** 1 Advanced Materials Metrology and Life Science Division, INRiM (Istituto Nazionale di Ricerca Metrologica), Strada delle Cacce 91, Torino 10135, Italy; 2 Department of Applied Science and Technology, 19032 Politecnico di Torino, C.so Duca degli Abruzzi 24, Torino 10129, Italy; 3 Physikalisch Technische Bundesanstalt, Abbestr. 2-12, Berlin 10587, Germany; 4 Helmholtz-Zentrum Berlin, Hahn-Meitner-Platz 1, Berlin 14109, Germany; 5 CNR-SCITEC, via A. Corti 12, Milano 20133, Italy; 6 Università del Piemonte Orientale “A. Avogadro”, V.le Teresa Michel 11, Alessandria I-15121, Italy; 7 CNR-IMM, Agrate Brianza Unit, via C. Olivetti 2, Agrate Brianza 20864, Italy

**Keywords:** 2D memitter, neuromorphic computing, block copolymers, self-assembly, tungsten disulfide, adaptive photoluminescence

## Abstract

All-optical neuromorphic devices based on adaptive two-dimensional (2D) materials have the potential for mimicking the complex processing and memory capabilities of biological synapses. Recent research demonstrated synaptic plasticity and visual memory in WS_2_ monolayer-based 2D memitters (i.e., an emitter with memory). However, improving their optical performances is crucial for extending their scalability. Since the neuromorphic functionalities of 2D memitters relies on O_2_ and H_2_O desorption/absorption on WS_2_, a careful balance between photoluminescence intensity and surface preservation is critical. Here, we investigate the enhancement of time-dependent photoluminescence response, achieved through coupling WS_2_ flakes with plasmonic nanoparticles obtained by liquid phase infiltration of gold in self-assembled block copolymer micelles. The localized surface plasmon resonance of gold nanoparticles amplifies the electric field and improves light–matter interactions. This method enhances the 2D memitter optical properties while preserving its adaptive photoluminescence response, thus enabling neuromorphic behavior under optical stimuli.

## Graphical abstract

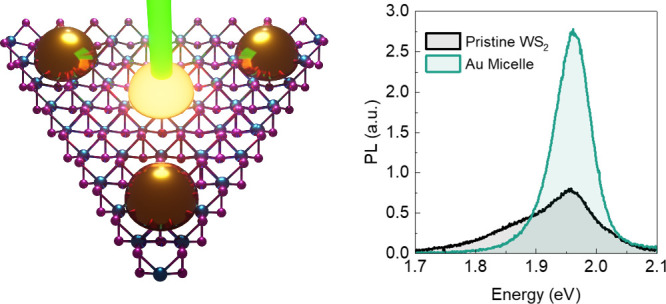

## Introduction

The growing interest in the optical and electrical properties of two-dimensional (2D) transition metal dichalcogenides (TMDs) arises from their unique electronic band structures, strong light–matter interactions at the atomic level, and mechanical flexibility. All these characteristics are highly suitable for a wide range of applications, including optoelectronics,^1–4^ flexible electronics,^5–7^ and energy harvesting,^7,8^ and in the realization of non-Von Neumann architectures.^9–11^ Besides that, TMDs and their heterostructures have also shown great potential in replicating neuromorphic processes typically observed in biological synapses. This capability has been successfully demonstrated using both purely electronic devices^12,13^ and optoelectronic systems.^14–16^ Recently, monolayer WS_2_ has found application in the realization of all-optical devices capable of mimicking processes of biological systems, such as short-term synaptic plasticity and visual short-term memory.^17^ The working principle of this device, termed a “2D memitter” (i.e., emitter with memory), relies on the time-dependent adaptive photoluminescence (PL) response of WS_2_ flakes under ambient conditions. Such a behavior is characterized by highly nonlinear dynamics, fading memory characteristics, and large-area synaptic response. These properties are directly linked to the inherent chemical composition of the WS_2_, as they are governed by adsorption and desorption processes of H_2_O and O_2_ on reactive sites, making the WS_2_-based memitter work under ambient conditions. This process results in a decrease of the characteristic n-doping in WS_2_ due to electron transfer to the adsorbed molecules, which in turn leads to an enhancement of the PL intensity over time.^18^ However, the low optical absorption cross section of monolayer WS_2_ related to its atomic thickness presents a significant challenge for the improvement of 2D memitter functionalities, resulting in relatively low quantum yield (∼6%, the highest among the TMDs) compared to other emitters like organic dyes,^19^ nanocrystals,^20,21^ and quantum dots.^22,23^ Strategies like encapsulation in organic polymers^24–26^ or coupling to photonic structures^27–29^ have been extensively explored as a method to tailor the PL emission of TMDs. Although these approaches successively improve the optical performances, they also introduce severe hurdles in the optimization of the functional properties of 2D memitters. Specifically, encapsulation and engineering of multilayered structures modify the surface of WS_2_, disrupting the crucial desorption/adsorption processes of H_2_O and O_2_ on reactive sites. This modification might alter the ability to replicate the key neuromorphic behavior typical of a 2D memitter. Therefore, enhancing the optical performances of WS_2_ for bioinspired devices, while maintaining its neuromorphic functionality, requires a careful balance between improving its PL intensity and preserving its pristine surface conditions. Over the years, various plasmonic structures, including those based on metals and dielectrics, have been proposed to enhance the PL intensity of TMDs.^30,31^ However, many of these structures involve complex fabrication procedures, often requiring multiple lithographic steps or transfer of TMDs from one substrate to another, which may alter their optical performances. An alternative strategy consists in coupling TMDs with plasmonic nanoparticles (NPs) such as Au or Ag nanorods,^32–34^ nanoprisms,^35^ nanoshells,^36,37^ and nanocubes.^38,39^ These NPs can support highly confined surface plasmons, facilitating room temperature energy and charge transfer with excitons^40,41^ and reversible phase transitions in 2D TMDs.^42^ While these plasmonic structures significantly enhance the PL, they often suffer from poor spatial uniformity when coupled with TMD flakes, resulting in very localized PL randomly located on the substrate area.^43^

In this work, we propose a different integration strategy by employing self-assembled gold (Au) NPs fabricated via liquid phase infiltration (LPI)^44–47^ in a polystyrene-*block*-poly­(2-vinylpyridine) (PS-*b*-P2VP) block copolymer (BCP). This templated approach enables the formation of spatially uniform arrays of AuNPs integrated onto monolayer WS_2_ flakes grown by chemical vapor deposition (CVD). The use of BCP not only provides a nanoscale template for AuNPs positioning but also ensures compatibility with large-area processing. A comprehensive characterization of the AuNPs, conducted at the local scale using atomic force microscopy (AFM) and scanning electron microscopy (SEM) and at the large scale using grazing incidence X-ray scattering (GISAXS), enables accurate modeling and simulation of the system’s optical response. Crucially, this method effectively enhances the overall PL intensity of WS_2_ flakes while preserving the synaptic functionalities of the 2D memitter, something that is not attainable with previously reported plasmonic architectures. By combining plasmonic nanostructures with 2D TMDs, this approach offers a scalable and device-compatible approach for enhancing and controlling the optical response of 2D materials in neuromorphic optoelectronic platforms.

## Results

### AuNP Formation over WS_2_ Flakes

The synthesis of WS_2_ flakes was carried out through CVD at 850 °C. To facilitate the growth process, a perylene-based molecule, *N*,*N*-bis­(5-guanidyl-1-pentanoic acid)-perylene-3,4,9,10-tetracarboxylic acid diimide (PTARG) (Figure 1a,b), was employed as a molecular seeding agent.^17^ PTARG controls the reaction kinetics, enabling the production of larger triangular flakes with dimensions exceeding 100 μm per side, as shown by the optical image in Figure 1c. Raman spectroscopy was used to confirm the monolayer nature of the WS_2_ flakes, with peak ratio analysis of the 2LA­(M) and A_1g_(G) modes (Figure 1d). The specific intensity ratio between these peaks is a well-established signature of monolayer WS_2_, as the vibrational modes exhibit shifts and intensity changes characteristic of a single atomic layer.^48^

**Figure 1:**
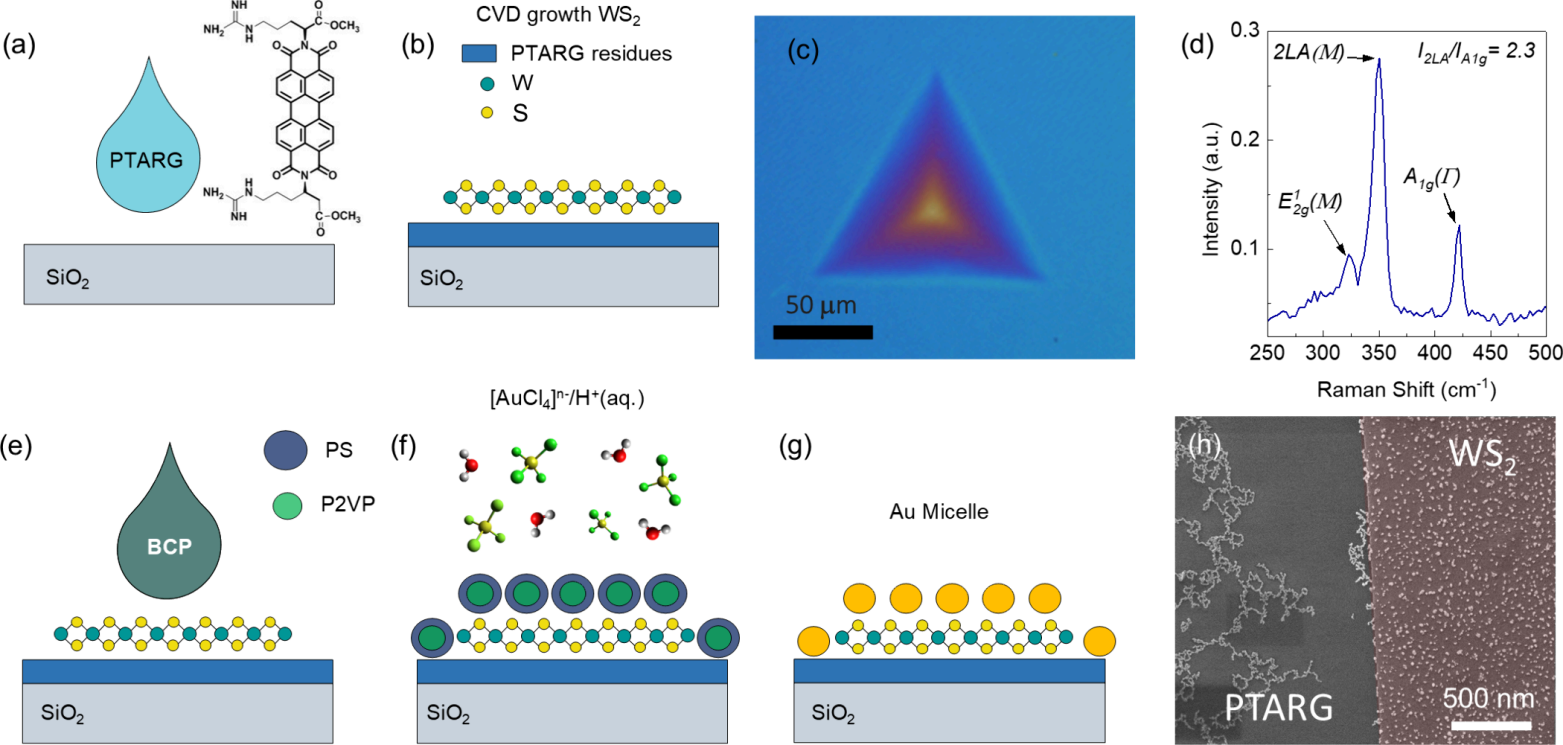
AuNP-based 2D memitter fabrication procedure. (a) Spin coating of PTARG in aqueous solution. (b) CVD growth of WS_2_ onto a Si substrate covered by 50 nm SiO_2_ and (c) optical image of the resulting large-area flakes. (d) Raman spectrum confirming the monolayer thickness of the CVD grown WS_2_. (e) Deposition of the PS-*b*-P2VP BCP solution by spin coating onto the WS_2_ flakes, enabling the formation of nanometric micelles. (f) Selective metal inclusion into micellar BCP by the LPI process. (g) Metal reduction by Ar plasma and formation of AuNPs. (h) SEM micrograph showing the deposition of the formation of AuNP clusters over the substrate and their homogeneous distribution on the WS_2_ flakes.

The Au nanoparticles (AuNPs) were synthesized using the BCP self-assembly method. A 9 mg/mL solution of PS-*b*-P2VP BCP in toluene was spin-coated onto the substrate on which the WS_2_ flake growth was performed (Figure 1e). This procedure leads to the formation of BCP micelles on the substrate without the need for additional thermal processes, as required for the formation of cylindrical or lamellar BCP structures. The selective incorporation of Au into micelles was obtained by the LPI method.^49^ This process consists of the selective electrostatic interaction of [AuCl_4_]^−^ anions to the pyridine moiety of P2VP, which is protonated after immersion of the self-assembled BCP micelles into an aqueous solution of the Au metal salt precursor (HAuCl_4_) and HCl. Subsequently, the sample is exposed to an Ar plasma, which reduces the Au salt localized within P2VP and simultaneously etches the BCP (Figure 1g), consequently leading to the formation of AuNPs. The SEM micrograph shown in Figure 1h illustrates the distribution of AuNPs on the WS_2_ flakes. Interestingly, the different wettabilities of the substrate and WS_2_ surfaces lead to an uneven location of AuNPs across the sample. It should be pointed out that the substrate after the growth is unavoidably affected by the residual components (mostly perylene aromatic cores) of the PTARG seeding promoter.^50^ While the hydrophilicity of the WS_2_ flakes promotes the homogeneous formation of AuNPs,^51^ the high processing temperature of the PTARG could increase its hydrophobicity of the substrate outside the flakes, thus resulting in the formation of AuNP clusters (Figure 1h and Figure S1).

### AuNP Characterization

The plasmonic behavior of the localized surface plasmon resonances (LSPR) in AuNPs is strongly influenced by their geometric size and shape. While their shape determines the number of LSPR supported by the system, the AuNP size governs their spectral position and the level of scattering and absorption of the incident radiation.^52–54^ Specifically, the LSPR observed in spherical NPs with sizes below 100 nm exhibit a strong absorption peak in the visible spectrum due to the coherent oscillation of conduction electrons.^55^ Additionally, in the sub-30 nm range, the scattering component is minimal, resulting in higher absorption efficiency if compared to scattering contribution.^56,57^ It is thus clear that the determination of both the size and shape of the BCP based AuNPs is fundamental for understanding their influence on the optical properties of WS_2_. Preliminary statistical analysis on SEM micrographs (Figure 2a) revealed a broad size distribution of the AuNPs centered around 20 ± 4 nm (Figures 2b). AFM measurements were performed to determine the height of the AuNPs, revealing a height distribution ranging from 7 to 15 nm (Figure 2c and Figure S2). However, SEM and AFM micrographs provide only partial and local information about the in-plane morphology of AuNPs, making it insufficient for a fully reliable characterization of the shape of plasmonic NPs over a large scale. Indeed, the selective infiltration and plasma reduction of Au salt precursors may significantly alter the initial spherical morphology of the micelles (see Figure S3) and consequently their optical behavior. To gain a comprehensive understanding of the morphology, we performed GISAXS analysis of the AuNPs. In order to disentangle possible contributions of the WS_2_ flakes and the Au clusters, the NPs were dispersed over a bare SiO_2_ substrate. More details about the GISAXS analysis are reported in the Experimental Section. To gain more insights about the 3D morphology of the AuNPs, the GISAXS scattering map of the BCP micellar system was simulated and fitted using various form factors and tailored geometric parameters provided by *BornAgain* software.^58^ The comparative analysis between experimental results and simulations (Figure S4) highlights the presence of a characteristic Bragg peak at *q*_*x*_ = 0.12 nm^–1^, confirming the presence of AuNPs with an average interdistance of 53 nm, and the horizontal line cuts shown in Figure 2d suggests that the AuNPs have a hemispherical shape, with average diameter *d* = 16 nm and a height of *h* = 12 nm (Figure 2e). The observed shape deviates significantly from the expected spherical morphology of the original micelles, likely due to the collapse of the initial spherical structure (Figure S3) upon plasma removal of the polymer component. To contribute to the evaluation of the plasmonic behavior of the micelles, near-edge absorption fine structure (NEXAFS) analysis was performed, allowing the determination of the oxidation state of gold following the LPI process in the micelles on a bare silicon substrate. Figure 2f reports the NEXAFS spectra acquired on the micelle sample and on a reference homogeneous layer. The gold L_III_ absorption edge is marked at 11.919 keV. The two characteristic features of the Au(0) spectrum at 11.95 and 11.97 keV are highlighted in the reference spectrum by the dashed lines, as previously reported.^59^ The presence of the same features in the micelle spectrum clearly indicates that gold in its elemental state is formed as a result of the infiltration of HAuCl_4_ and the reduction by Ar plasma.

**Figure 2:**
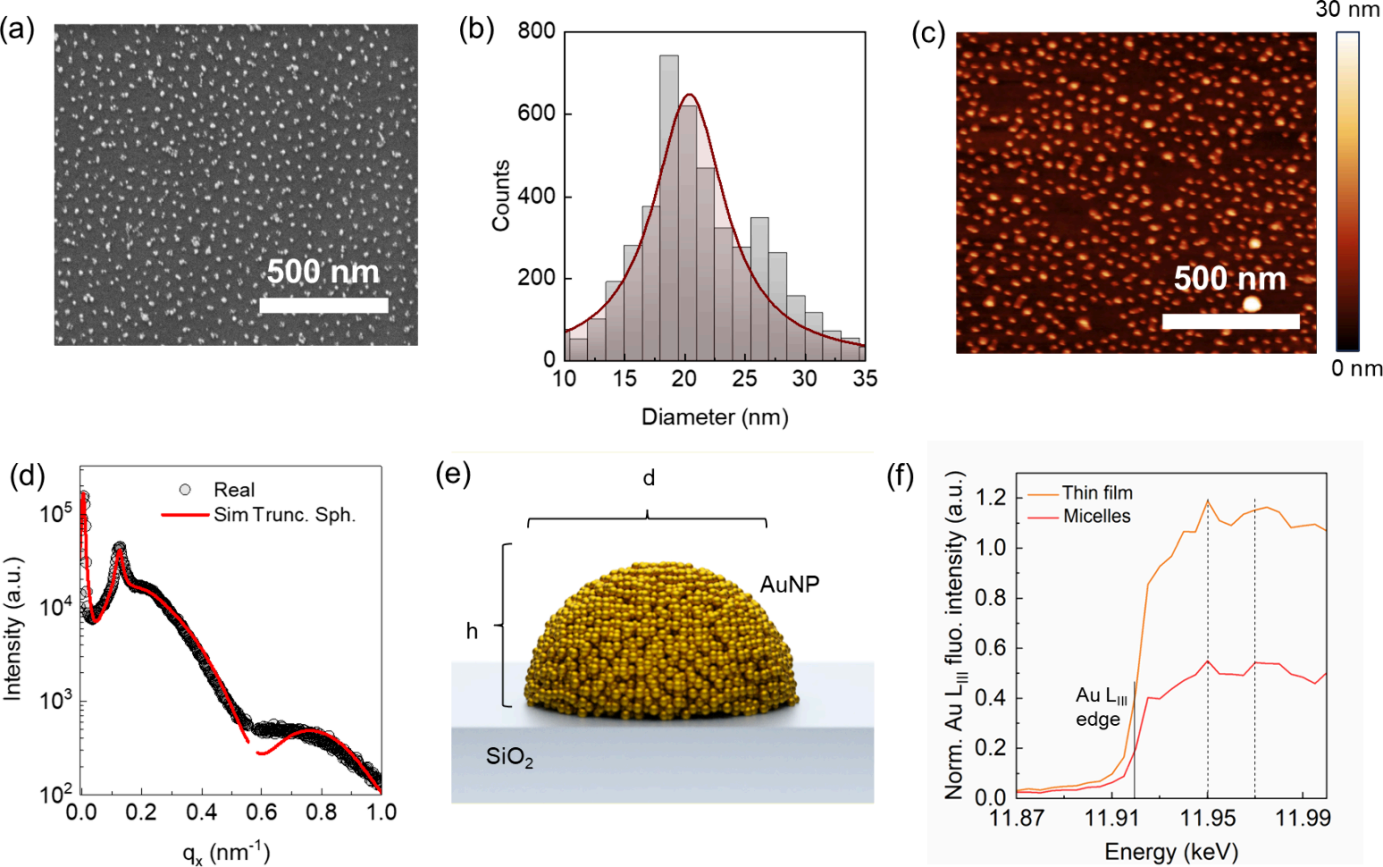
NP characterization. (a) SEM micrograph showing the distribution of the AuNPs over WS_2_ and on the substrate. (b) Diameter distribution of the AuNPs calculated by SEM images. (c) AFM map describing the height distribution of the AuNPs. (d) The horizontal line cut on 2D GISAXS data of the nanostructured sample provides information concerning the form factor, diameter, and height of the NPs. Red lines represent the experimental data fit extracted from simulations. (e) Schematic representation of the AuNP with a hemispherical shape. (f) NEXAFS spectra of a gold homogeneous film and micelles obtained by liquid phase infiltration.

### Optical Properties of AuNPs Coupled to WS_2_

Besides the geometry of the NPs, the interaction of electromagnetic (EM) radiation with plasmonic nanostructures is significantly influenced by the surrounding dielectric environment.^60^ In particular, the presence of a nearby substrate induces an asymmetry in the system modifying the energy of plasmon modes and inducing a polarization-dependent degeneracy of the modes.^61^ In this context, finite element (FE) simulations were conducted in order to investigate the effect of the substrate and the presence of the 2D WS_2_ on the optical behavior of the hemispherical AuNPs. A key parameter useful to describe the optical response of the AuNPs is the normalized extinction cross section (β_ext_), which quantifies the proportion of light that is scattered or absorbed by the AuNPs as a function of the incident photon energy. β_ext_ can be defined as

$$\beta_{\mathrm{ext}}=\sigma_{\mathrm{ext}}/\pi r^{2}$$ 1

where σ_ext_ is the extinction cross section, normalized over the NPs area (π*r* ^2^). Notably, a resonant behavior in β_ext_ is observed when AuNPs with diameter within the range identified in the morphologic analysis (between 10 and 30 nm) are placed on the 2D WS_2_ compared to the same AuNPs placed on a bare SiO_2_ substrate (Figure 3a). This leads to a strong enhancement of the EM field distribution (calculated at 1.9 eV), especially underneath the NPs coupled to the 2D WS_2_ layer (Figure 3b) with respect to that of the AuNPs placed over a conventional SiO_2_ substrate (Figure 3c). The spectral position of the β_ext_ peaks, which are influenced by the LSPR, can be finely tuned by varying the particle size, up to a maximum value of β_ext_ = 120 for NP diameter *d* = 25 nm and height *h* = 12 nm (Figure 3d). On the other hand, a limited increase in β_ext_ is observed for AuNPs with *h <* 12 nm, as reported in Figure S5. Interestingly, the maximum β_ext_ value perfectly matches the PL spectrum typical of the 2D WS_2_ (black line in Figure 3e). The reported increase in β_ext_ results in an overall PL enhancement when WS_2_ is coupled with the plasmonic NPs (Figure 3f). The PL spectrum coupled with the NPs exhibits a narrower fwhm compared with that of pristine WS_2_. Furthermore, the broad size distribution of the AuNPs in both diameter and height, reported in Figure 2c and Figure S3, prevents the appearance of distinct peaks in the PL spectrum corresponding to the extinction cross section. Additionally, the work function (WF) difference between the AuNPs (∼5.1 eV)^62^ and WS_2_ (∼3.5 eV), measured by scanning Kelvin probe force microscopy (KPFM) and reported in Figure 3g, plays a crucial role in facilitating hot carrier transfer under laser irradiation at 2.38 eV. Indeed, hot electrons in the AuNPs, generated through plasmonic excitation, can overcome the Schottky energy barrier (typically in the range 1.1–1.6 eV for n-type WS_2_) varying from a complete Fermi-level pinning configuration to an ideal Schottky–Mott behavior for degenerate semiconductor^63^ and transfer energy to the conduction band of WS_2_. This hot carrier injection process can further enhance the PL by increasing charge separation at the metal–semiconductor interface, which leads to a more efficient radiative decay of excitons.^41^

**Figure 3:**
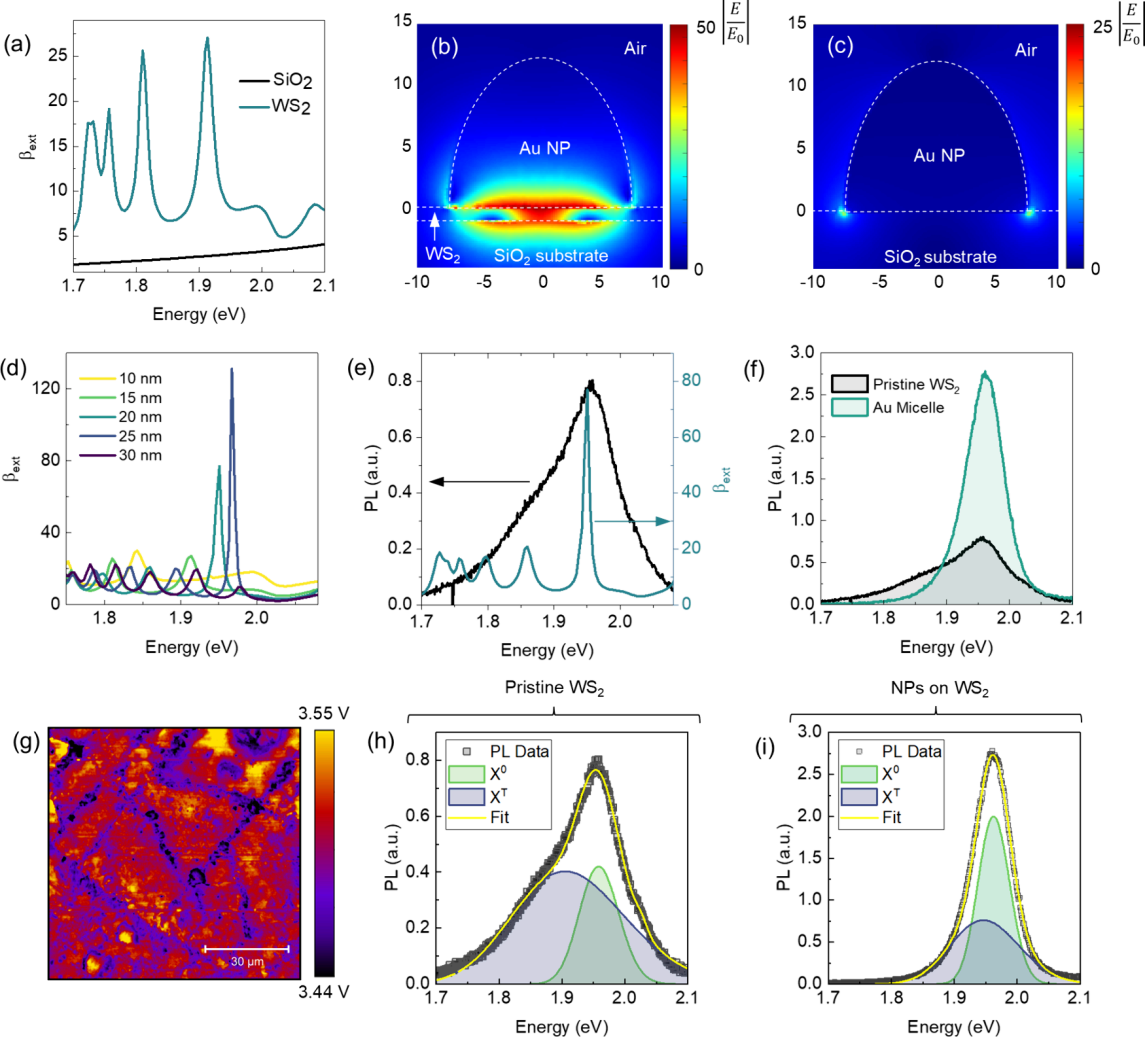
Plasmonic enhancement. (a) Normalized extinction cross section (β_ext_) of a 15 nm-diameter AuNP placed on a SiO_2_ substrate (black curve) and on a WS_2_ flake (green curve). Corresponding distribution of the EM field around a AuNP (calculated at β_ext_ = 1.92 eV) placed over (b) a pristine SiO_2_ substrate and (c) a WS_2_ layer. (d) β_ext_ values for AuNPs with diameter ranging between 10 and 30 nm. (e) The PL spectrum of a pristine WS_2_ flake (black curve) overlapped with the β_ext_ of AuNP with a 20 nm diameter (green curve). (f) PL spectra measured on a pristine WS_2_ flake and on the WS_2_ coupled to the self-assembled AuNPs. (g) WF map of WS_2_, measured by scanning KPFM. The scale bar is 30 μm. Gaussian peak deconvolution showing the contribution of X^0^ (green curves) and X^–^ (blue curves) on the PL of a (h) pristine WS_2_ flake on SiO_2_ and (i) WS_2_ coupled with AuNPs.

The enhanced electromagnetic field near the WS_2_ layer driven by the LSPR increases the absorption cross section of the AuNPs. This results in an enhancement in both the dipole–dipole Foster resonance energy transfer (FRET) scheme and the Dexter charge transfer process, i.e., hot carrier injection in WS_2_, providing additional carriers for recombination. Charge transfer is typically active when the two materials are in close proximity (typically below 1 nm), while in 2D materials, FRET energy transfer efficiency rescales with the junction spacing *d* as 1/*d* ^4^; therefore, a superposition of both effects is expected in our system.^64,65^ Both significantly alter the shape of the PL spectrum, thereby providing a way for filtering the exciton population in TMDs at room temperature and under ambient conditions. In particular, in the case of n-type WS_2_, the two mechanisms cooperate, affecting the PL spectrum with a similar phenomenology. Specifically, this interplay enhances the number of neutral excitons (X^0^) relative to trions (X^–^) in the PL spectrum.

The modification is validated through Gaussian deconvolution of the PL signal, which identifies the contributions from X^0^ and X^–^ in both pristine 2D WS_2_ (Figure 3h) and WS_2_ coupled with AuNPs (Figure 3i). The injection of hot carriers enhances the radiative decay rate of X^0^, leading to a significant enhancement in PL intensity, further amplified by the plasmonic near-field effects from the AuNPs. Overall, the combined influence of the enhanced EM field and efficient hot carrier transfer due to the WF mismatch significantly boosts the optical performance of the WS_2_-AuNP system. As demonstrated in recent experiments at variable temperatures,^24^ elevated temperatures lead to an increase in the thermal dissociation of X^–^, resulting in a consequent decrease in the trion-to-exciton ratio.

### Stimuli-Responsive PL in 2D Memitters

WS_2_ flakes have been demonstrated to function as 2D memitters, enabling the all-optical implementation of neuromorphic functionalities.^17^ We refer to the memitter as a material or a device whose light emission is influenced by its history of exposure to optical stimuli.^66,67^ In this context, short-term synaptic plasticity and visual short-term memory functionalities typical of biological systems can be emulated by exploiting the intrinsic capability of the system to nonlinearly adapt to its internal state over time (i.e., PL response) depending on the input stimulation (i.e., laser pulse) followed by spontaneous relaxation to the ground state after the end of stimulation (Figure 4a). In the following, we show that the proposed strategy for enhancing the PL of WS_2_ can be exploited in the 2D memitter since this does not interfere with its adaptive response to optical stimulation. Synaptic functionalities in WS_2_ flakes coupled to AuNPs were achieved using the optical scheme depicted in Figure 4b: the potentiation stimulus consists of a high-power density (i.e., 1 mW/μm^2^) continuous-wave (CW) laser irradiation, while the spontaneous relaxation is probed by monitoring the PL in a pulsed wave (PW) regime.^17^ As a result of this experiment, we observed an increase in PL intensity over time under CW irradiation (Figure 4c), highlighting the ability of the 2D memitter to exhibit synaptic-like potentiation. A decay over time of the PL intensity is then observed in the PW regime, as reported in Figure 4d. The mechanism underlying this behavior is related to the desorption/absorption process of O_2_ and H_2_O over WS_2_. Analyzing the evolution of the PL spectrum intensity peaks over time (black squares in Figure 4e), the characteristic behavior of 2D emitters is observed. This consists of an initial enhancement phase followed by a relaxation phase of the PL, in response to PW stimulation. Following an analogy with biological systems, the PL response of the device (governed by the internal state of the 2D memitter) acts as the synaptic weight, with its dynamics determined by the history of the applied stimulation. These dynamics are fundamentally linked to the adsorption and desorption processes of O_2_ and H_2_O at the WS_2_ surface, which typically follow rate-limited kinetics. Such mechanisms naturally give rise to exponential time dependencies in the PL signal. In order to provide a numerical comparison between the PL rise and decay rates in the pristine 2D memitter and the WS_2_-AuNP memitter system, we assessed the potentiation (τ_a_) and depression (τ_b_) time constant by fitting the PL intensity of the two systems with a single exponential growth/decay curve. Interestingly, we observe that both τ_a_ and τ_b_ are significantly faster in the presence of AuNPs. Specifically in the case of the WS_2_-AuNP memitter, τ_a_ ≈ 187 ms and τ_b_ ≈ 105 ms, and for the pristine WS_2_ memitter, τ_a_ ≈ 258 ms and τ_b_ ≈ 894 ms.

**Figure 4:**
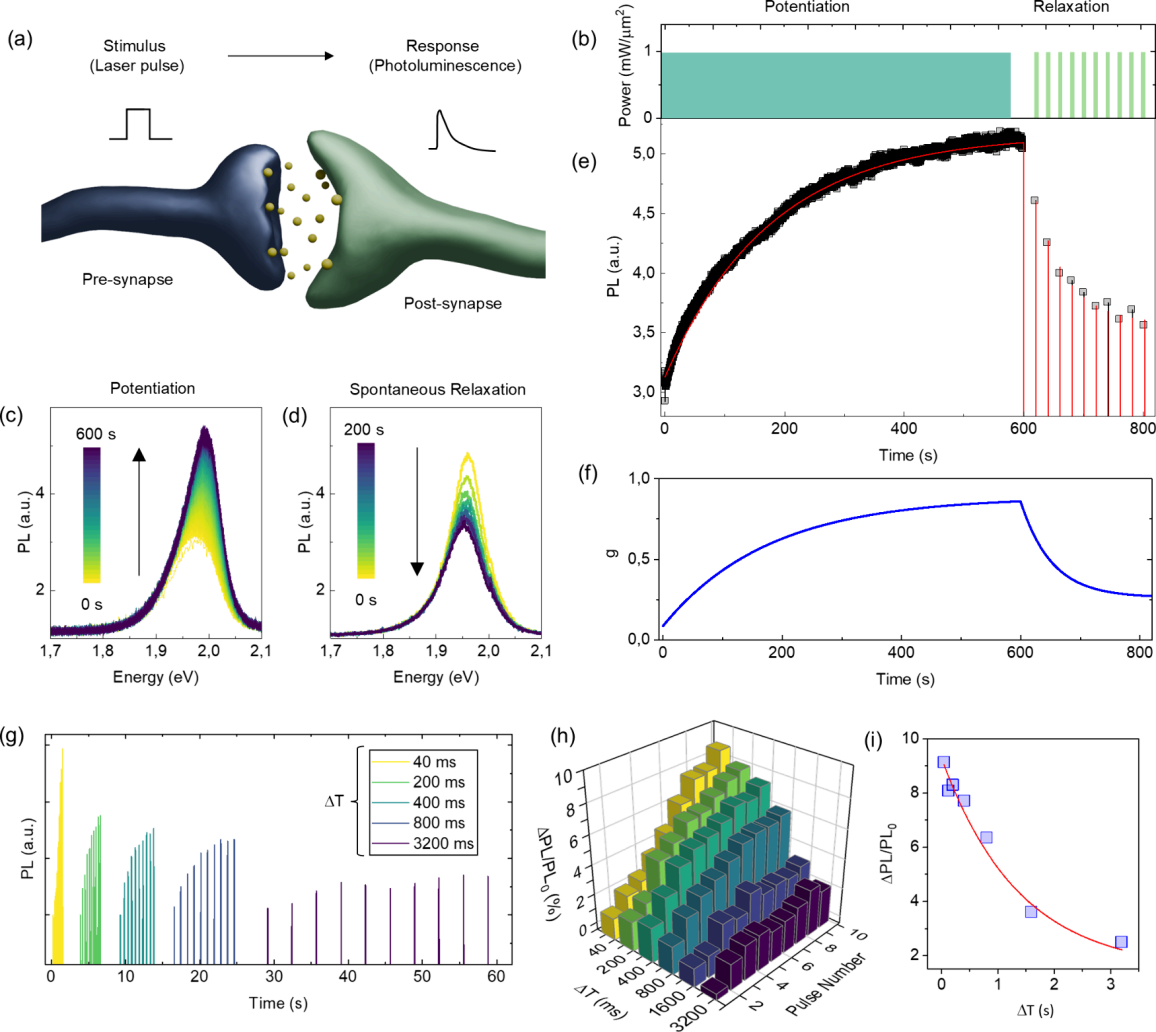
Adaptive PL response. (a) Schematic model representing a biological synapse, in which a nanometric cleft separates the presynapse and postsynapse. Upon receiving stimuli, the presynaptic terminal releases the transmitters toward the postsynaptic terminals. The postsynaptic membrane owns numerous neurotransmitter receptors that have the potential to respond to the released transmitters, thereby facilitating the postsynaptic response. (b) 2D memitter excitation scheme, consisting of a CW potentiation process (600 s at 1 mW/μm^2^) followed by a 10 PW reading pulse (200 ms at 1 mW/μm^2^ with 20 s repetition rate). PL spectra as a function of time measured during the (c) potentiation and (d) spontaneous relaxation processes. (e) Maximum PL intensity values (black squares) recorded under the excitation schemes described in (b), overlapped on the model curve based on the potentiation–depression rate balance equation (red line). (f) Evolution of the normalized internal memory state *g* as a function of time of the WS_2_ memitter coupled to AuNPs. (g) Variation of the PL intensity under pulsed wave potentiation (pulse duration of 200 ms and variable pulse delay time between 40 and 3200 ms). (h) Bar plot describing the percentage increase in PL normalized to the intensity of the first pulse (PL_0_), plotted as a function of pulse delay (Δ*T*) and pulse number. (i) Variation of the normalized ΔPL of pulse number 10 as a function of Δ*T* (blue squares). The red line represents the exponential decay fit of the reported data.

To generalize the observed exponential trends, we adopt a more comprehensive model based on a rate balance equation, described in ref 17 (red curve in Figure 4e), to capture the complete dynamical response of the device. This model serves as a behavioral framework that generalizes the single exponential functions and allows for the simulation of both potentiation and depression dynamics under arbitrary stimulation histories. As shown in Figure 4f, this approach not only allows interpolation of experimental data but also enables modeling and evaluation of the internal memory state *g* dynamics even in the absence of external stimulation.

As already observed when characterizing the pristine 2D memitter,^17^ the potentiation process can be driven not only in CW mode but also through a series of consecutive pulses. Figure 4g reports the PL variation of the AuNP–WS_2_ coupled system when stimulated with a series of 10 pulses having fixed duration of 200 ms and variable interval between consecutive pulses Δ*T* between 40 and 3200 ms. The bar plot reported in Figure 4h, describing the percentage increase in PL normalized to the intensity of the first pulse (ΔPL/PL_0_), shows a decrease of the ΔPL/PL_0_ when increasing Δ*T*. This is further testified by exponential decrease of the ΔPL/PL_0_ after 10 pulses when increasing Δ*T* (reported in Figure 4i).

This behavior demonstrates that the AuNP–WS_2_ coupling does not interfere with the inherent nonlinear PL dynamics of the memitter, which maintains its peculiar characteristics such as the all-optical short-term synaptic plasticity.

## Conclusions

In conclusion, we demonstrate the possibility of enhancing the PL of WS_2_-based 2D memitters through plasmonic coupling induced by AuNPs. The use of BCP self-assembly to fabricate AuNPs enables a straightforward method to achieve a homogeneous nanoparticle distribution on WS_2_ without requiring complex lithographic steps and therefore is highly and cheaply scalable to the wafer scale. Morphological characterization of the AuNPs, performed using lab-based techniques and X-ray scattering, along with FE simulations, allowed us to understand the behavior of the electromagnetic field in the vicinity of the AuNPs coupled with the WS_2_ memitter. As a result of this fabrication process, we achieved an increase in PL in WS_2_ coupled with AuNPs compared to that of pristine WS_2_. Notably, the PL enhancement does not impair the neuromorphic functionalities of the memitter, which include an increase in PL over time when the device is subjected to CW irradiation and spontaneous relaxation when stimulated in PW mode. Overall, the coupling of AuNPs appears to boost the memorizing–forgetting speeds of the optical inputs of the WS_2_-based memitter, offering a tunable platform for temporal information processing at faster time scales than pristine WS_2_. This type of system paves the way for the development of a new class of all-optical neuromorphic devices capable of replicating synaptic-like behavior. Such devices could lead to advancements in optical computing, where efficient, nonelectronic mechanisms for data processing and memory storage are increasingly desirable.

## Experimental Section

### Materials and Methods

#### Synthesis of WS_2_ Crystals

WS_2_ flakes were synthesized in a CVD reactor using a hot-wall furnace and a 1 in.-diameter fused quartz tube. An alumina boat containing a mixture of 35 mg of WO_3_ (99.9%, Sigma-Aldrich) and 5 mg of NaCl (>99%, Sigma-Aldrich) powders was placed at the center of the tube, while the growth substrates, a 50 nm-thick SiO_2_ layer over Si^2+^, were positioned approximately 10 mm above the powder, with the polished side facing downward. Another boat with about 100 mg of sulfur powder (99.98%, Merck) was positioned in the inlet region of the quartz tube. Prior to loading into the CVD system, the substrates underwent a three-step cleaning process: 5 min in acetone and 5 min in 2-propanol, followed by 5 min of rinsing in deionized (DI) water. After cleaning, the substrates were dried using a nitrogen flow. To prepare the SiO_2_ substrate, a seeding promoter solution was applied. The seeding promoter PTARG was synthesized as follows: perylene-3,4,9,10-tetracarboxylic dianhydride (1.3 mmol) was combined with imidazole (29 mmol) and stirred vigorously at 110 °C. After this, 1.8 mmol of arginine methyl ester dihydrochloride was added to the mixture and stirred for 4 h, resulting in a dark-red solid precipitate as the reaction mixture cooled. The precipitate was washed several times with methanol to remove imidazole, yielding a dark-red solid with 75% yield. A PTARG solution (0.37 mg in 10 mL of distilled water) was used to prepare a 50 μm L^–1^ solution. This solution was applied to the 2 cm^2^ substrate surface by dropping 10 drops (approximately 50 mg) using a pipet. The CVD growth process was carried out by heating the furnace to 850 °C at a rate of 10 °C/min, with a carrier gas flow of 0.2 L h^–1^ (H_2_/Ar, 4% H_2_)

#### AuNP Fabrication

AuNPs were synthesized by liquid phase infiltration of BCPs.^43^ Specifically, 9 mg of PS-*b*-P2VP with a molecular weight (*M*_w_) of 199 kg/mol, a PS volume fraction (f_PS_) of 0.51, and a polydispersity index (PDI) of 1.12 (purchased from Polymer Source Inc. and used without further purification) were dissolved in 1 mL of toluene and spin-coated on the substrate without any surface functionalization and then immersed in an aqueous solution of HAuCl_4_ (40 mM) with HCl (9.25% w/w) for 10 min at room temperature to promote the polymer metal loading, followed by water rinsing. Afterward, a 10 min exposure of the loaded polymer to Ar plasma at 40 W led to the simultaneous polymer removal and metal ion reduction, revealing the AuNPs.

#### GISAXS Analysis

The morphological characterization of the AuNPs was carried out at the micro- and nanofocus small- and wide-angle X-ray scattering beamline (MiNaXS) P03 at PETRA III of DESY (Hamburg, Germany). The samples were measured at a grazing angle of α = 0.4° and a sample–detector distance of 4050 mm. The X-ray scattering patterns were recorded using a PILATUS detector with a single pixel area of 172 × 172 μm^2^. As reported in Figure S2, the experimental data were compared to fitted and simulated obtaining a circular cross section with different form factors (i.e., sphere, hemisphere, hemiellipse, and truncated sphere) assembled in a two-dimensional hexagonal lattice.^58^

#### NEXAFS Analysis

The NEXAFS experiments were carried out at the 7-T wavelength shifter (WLS) beamline BAMline^68^ at the BESSY II electron storage ring in fluorescence detection mode, probing the Au-L3 shell at a 45° incident angle with respect to the sample surface. At each photon energy, a fluorescence spectrum was recorded employing a Bruker XFlash silicon drift detector, from which the count rate for the Au-Lα fluorescence line was evaluated and normalized to the incident photon flux. The NEXAFS data shown in Figure 2f were acquired on a micellar PS-*b*-P2VP sample infiltrated with HAuCl_4_ in the liquid phase and on a homogeneous gold layer as a reference. Both curves were normalized to the last value of the reference data.

#### PL Measurement

The characterization of the responsive PL behavior was carried out using a confocal system equipped with a CW excitation laser operating at 520 nm (MatchBox Laser Diode from Integrated Optics), and focused by a 20× microscope objective, from where fluorescence is collected in an epifluorescence configuration. The laser light is filtered by an edge filter (FEL550 from Thorlabs). PL signal coming from the WS_2_ memitter is then coupled to a multimode fiber and sent to as SM-USB2000+ spectrometer from Ocean Optics for spectral analysis.

#### Scanning Kelvin Probe Force Microscopy

Scanning KPFM was performed using an Oxford Instruments Asylum Research MFP-3D atomic force microscope and Asylum Electrilever Ti/Pt-coated cantilevers (nominal resonance frequency *f* = 75 kHz; nominal spring constant *k* = 2.8 N/m; nominal tip radius *R* = 25 nm). Measurements have been performed in AC, two-pass mode (referred to as NAP mode), according to the procedure described in ref 62. Briefly, at each line, the scan is repeated two times to acquire topography, during the first pass, and with an elevation δ*z*, usually in the range 30–50 nm, to acquire contact potential between the tip and local features of the sample. The WF of the tip is calibrated on freshly cleaved highly oriented pyrolytic graphite (HOPG) reference (WF_HOPG_ = 4.6 eV with a variability of 4 mV_rms_, which defines the accuracy on the measurement) before measurements and used to retrieve the absolute value of the sample. Relative humidity during measurements and calibration is RH < 5% to avoid humidity contribution to the CPD measurement from the surface.

## Supplementary Material



## Notes

Description of the materials and methods, SEM and AFM micrographs of the AuNPs and infiltrated micelles, GISAXS analysis and simulated scattering patterns with different form factors, and simulation of the normalized absorption scattering cross section as a function of the AuNPs diameter and height (PDF)
