# Supplementary material for: Enhanced Photoluminescence in a Neuromorphic 2D Memitter Based on WS2 via Plasmonic Nanoparticle Self-Assembly
Source: ACS Appl Mater Interfaces. 2025 Jun 5;17(24):35695–704. doi: 10.1021/acsami.5c03059 (PMC12186229; doi:10.1021/acsami.5c03059)
Supplement: Supplementary file 1 [file am5c03059_si_001.pdf]

Supporting Information

Enhanced Photoluminescence in a Neuromorphic 2D Memitter based on WS_2 *via* Plasmonic Nanoparticles Self-Assembly

Federico Ferrarese Lupi,[1] Gianluca Milano,[1] Angelo Angelini,[1] Mateo Rosero-Realpe,[2] Irdi Murataj,[1] Bruno Torre,[1] Eleonora Cara,[1] Philipp Hönicke,[3,4] André Wählisch,[3] Erika Kozma,[5] Diego Antonioli,[6] Michele Laus,[6] Alessia Motta,[7] Christian Martella,[7] Carlo Grazianetti*[7]*

[1] Advanced Materials Metrology and Life Science Division, INRiM (Istituto Nazionale di Ricerca Metrologica), Strada delle Cacce 91, 10135 Torino, Italy.

[2] Department of Applied Science and Technology, Politecnico di Torino, C.so Duca degli Abruzzi 24, 10129 Torino, Italy.

[3] Physikalisch Technische Bundesanstalt, Abbestr. 2-12, 10587 Berlin, Germany

[4] Helmholtz-Zentrum Berlin, Hahn-Meitner-Platz 1, 14109 Berlin, Germany

[5] CNR-SCITEC, via A. Corti 12, Milano 20133, Italy

[6] Università del Piemonte Orientale ''A. Avogadro'', V.le Teresa Michel 11, Alessandria I-15121, Italy

[7] CNR-IMM, Agrate Brianza Unit, via C. Olivetti 2, 20864 Agrate Brianza, Italy

* Corresponding Authors: federico.ferrareselupi@inrim.it (FFL) and carlo.grazianetti@cnr.it (CG)

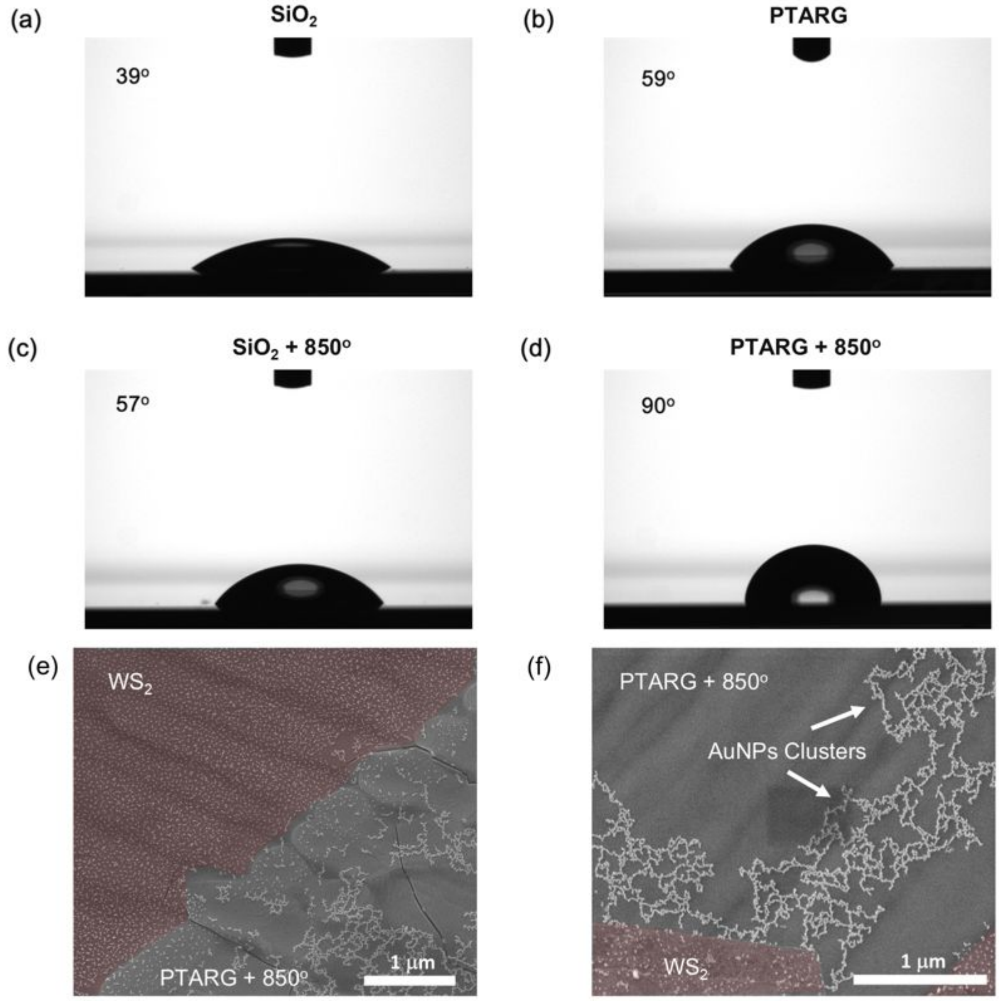

Figure S1. Water contact angles of the SiO_2 substrate after (a) piranha cleaning and (b) PTARG deposition. The same experiments were replicated after performing the thermal treatment at 850 °C respectively on (c) SiO2 substrate and (d) PTARG. (e) SEM micrographs describing the homogeneous distribution of the AuNPs over the WS_2 (marked in red) and (f) the cluster formation over the PTARG residuals (indicated by white arrows).

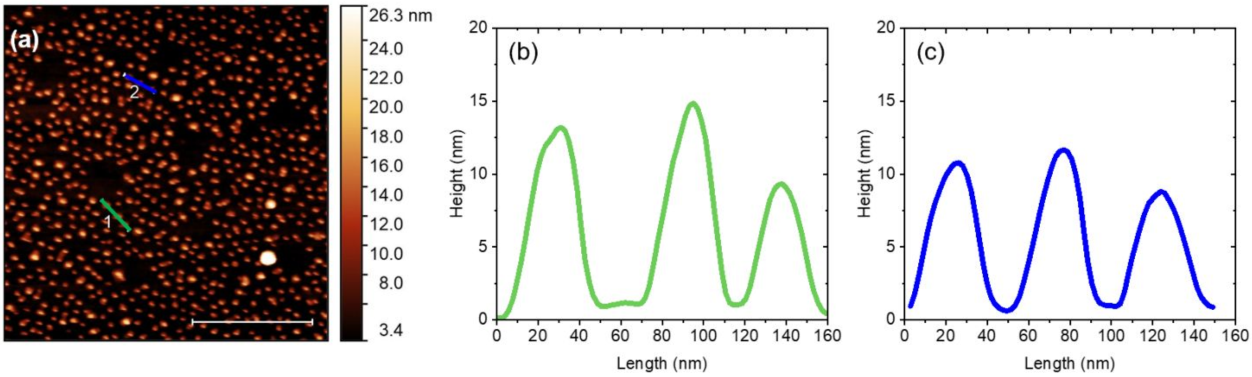

Figure S2. (a) AFM micrograph of the AuNPs. The height profiles extracted respectively in correspondence of the green line (b) and blue line (c) revealed the presence of NPs with height ranging between 7 and 15 nm.

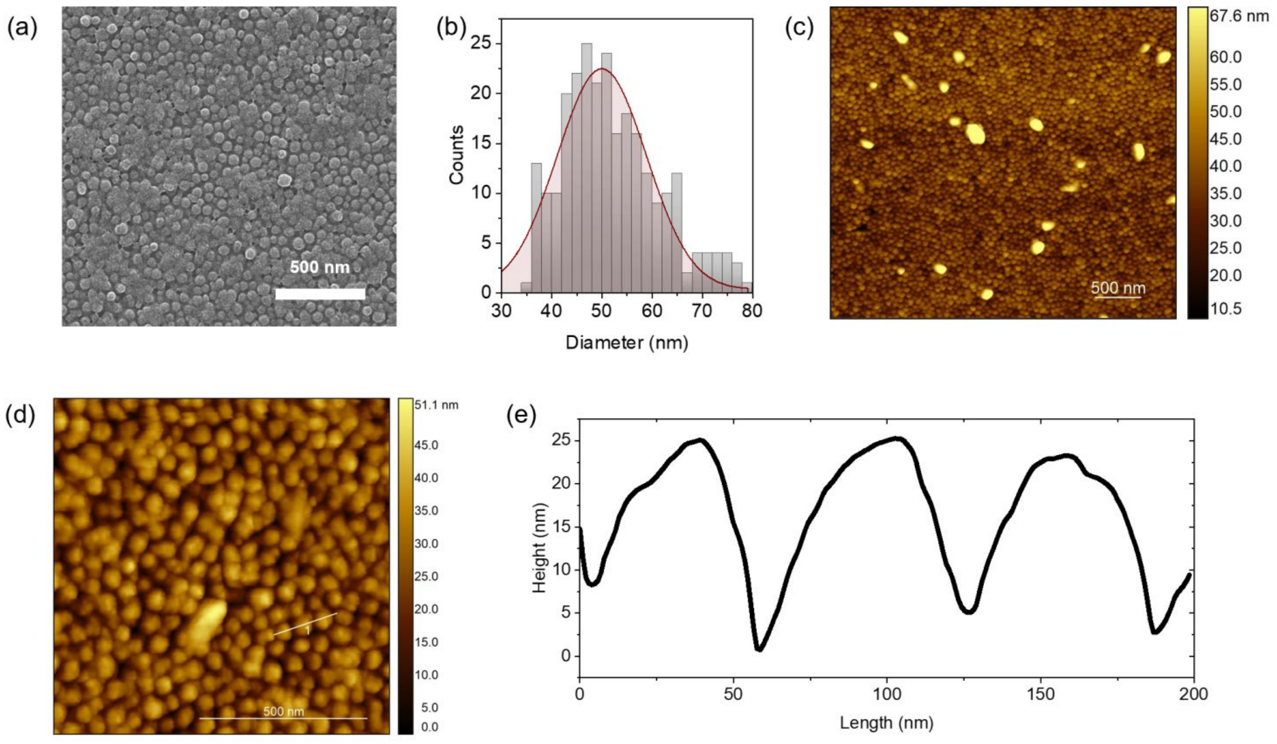

Figure S3. Morphologic characterization of the self-assembled micelles. (a) SEM micrograph of the self-assembled micelles before the plasma treatment and (b) corresponding diameter distribution. (c)-(d) AFM topography maps and (e) height profiles of the as-deposited micelle.

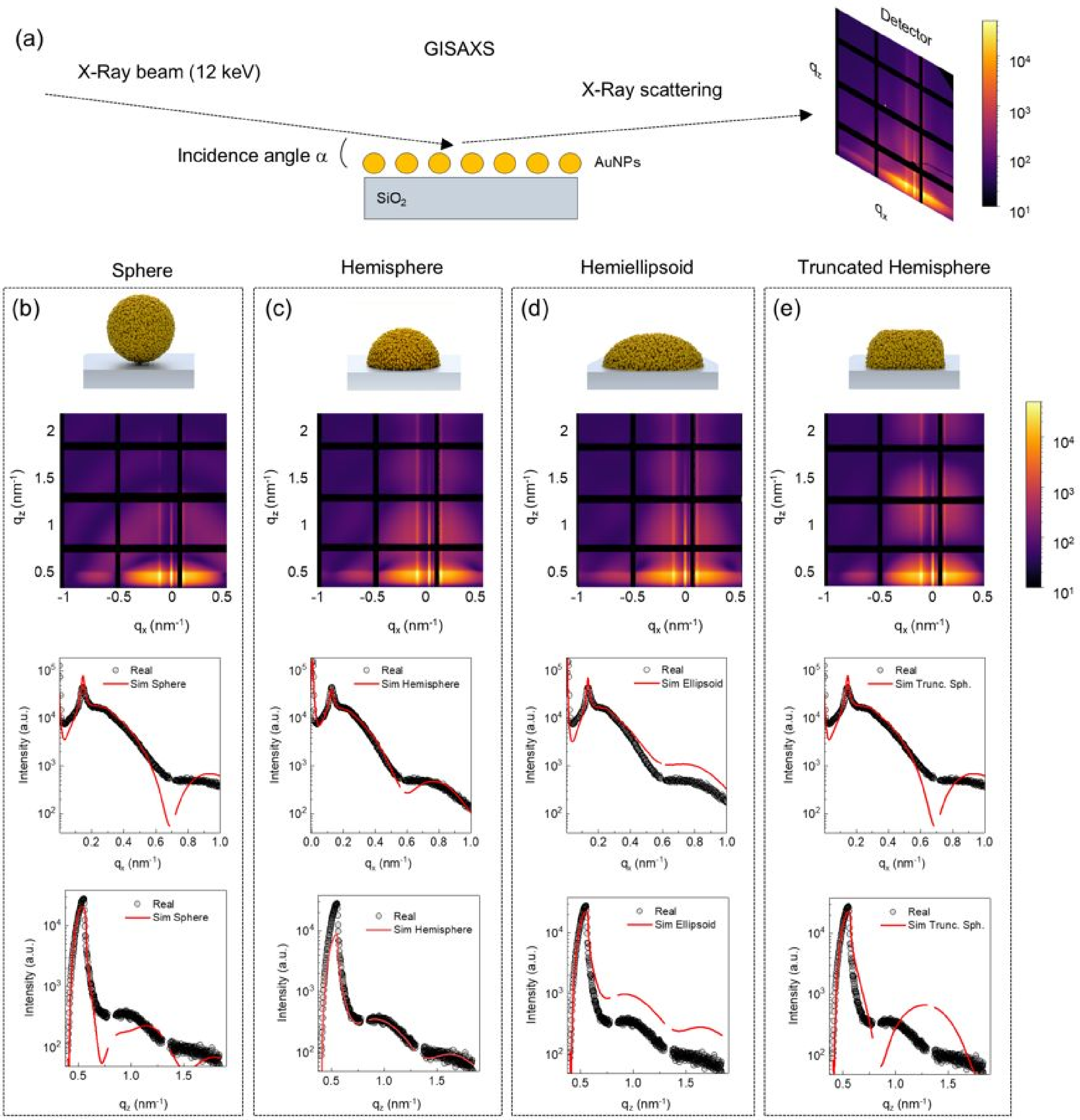

Figure S4. (a) Scheme of the experimental setup used in the GISAXS analysis of the AuNPs. The experimental data were compared to fitted and simulated obtained with different form factors: (b) sphere, (c) hemisphere, (d) hemiellipse and (e) truncated sphere. The intensity profiles along the horizontal and vertical axis were obtained by cutting the GISAXS scattering patterns at q_z = 0.5 nm^{-1} and q_x = -0.14 nm^{-1} respectively.

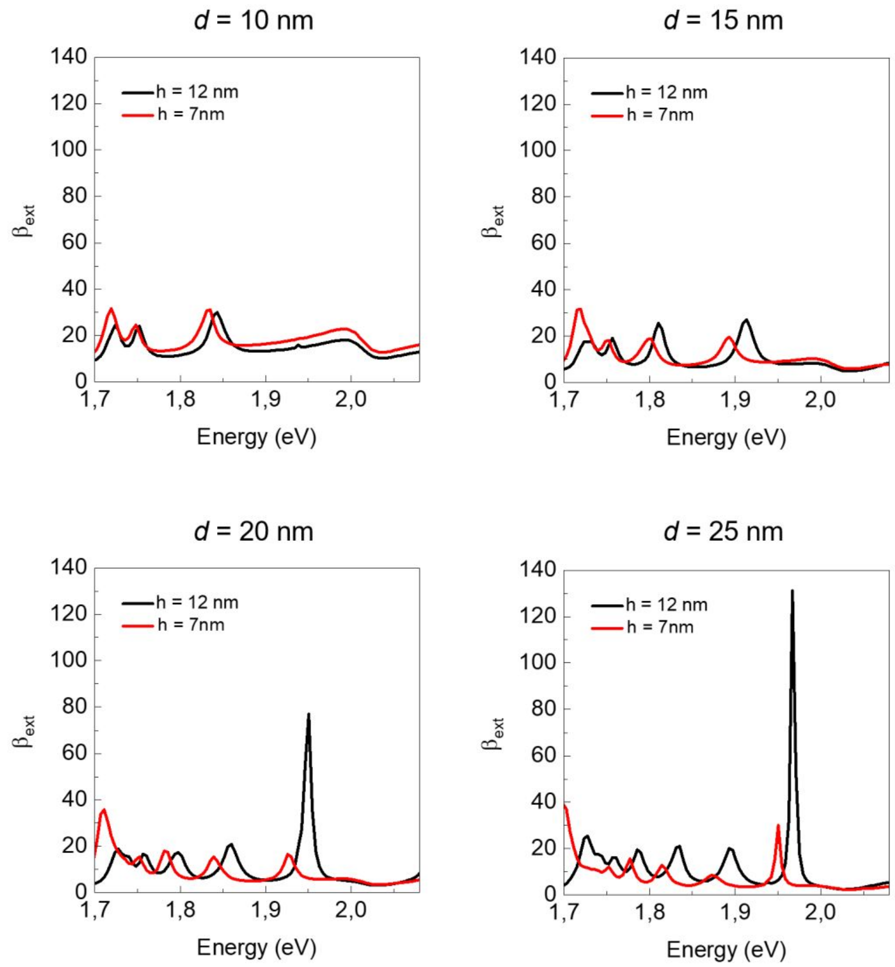

Figure S5. Normalized extinction cross-section (β_{ext}) of a AuNP on WS_2 having height 12 nm (black curve) and 7 nm (red curve) and diameter of (a) 10 nm, (b) 15 nm, (c) 20 nm and (d) 25 nm.
